# The effect of five versus two personnel on bacterial air contamination during preparation of sterile surgical goods in the operating room: a randomised controlled trial

**DOI:** 10.1186/s13756-025-01589-4

**Published:** 2025-06-15

**Authors:** Camilla Wistrand, Bo Söderquist, Ann-Sofie Sundqvist

**Affiliations:** 1 Department of Cardiothoracic and Vascular Surgery, Örebro University Hospital, O-house, 4th floor, Örebro, 701 85 SE Sweden; 2 University Health Care Research Centre, Faculty of Medicine and Health, Örebro University, Örebro, Sweden; 3 School of Medical Sciences, Faculty of Medicine and Health, Örebro University, Örebro, Sweden; 4 Department of Laboratory Medicine, Faculty of Medicine and Health, Örebro University, Örebro, Sweden; 5 Department of Orthopedics, Faculty of Medicine and Health, Örebro University, Örebro, Sweden

**Keywords:** Air quality, Contamination, Infection control, Microorganism, Operating room, Surgery, Surgical instrument, Surgical site infection

## Abstract

**Background:**

Surgical site infection (SSI) and antimicrobial resistance are a worldwide problem affecting patient safety. It is lacking randomised controlled trials (RCT) regarding how the number of personnel in the operating room (OR) affects the air quality. We aimed to investigate the effect the number of personnel in the OR have on bacterial air contamination during the preparation of sterile surgical goods, to identify the species and antibiotic susceptibility of the bacteria isolated, and to describe the number of SSIs together with causative microorganisms.

**Methods:**

This RCT used an intervention group in which two individuals prepared the surgical goods and a control group in which five individuals prepared the goods. Bacteria were isolated on aerobic and anaerobic plates, and bacterial growth was measured as colony forming units (CFU). All isolates were typed, and types known to cause SSI were tested for susceptibility to eight antibiotics. Data were analysed with the Mann-Whitney *U* test, the chi-square test, or Fisher’s exact test.

**Results:**

Results were based on 69 open-heart surgeries and 414 plates. When sterile surgical goods were prepared with two personnel, the median CFU was 2 with an IQR of 2, compared with five personnel, the median CFU was 5, with an IQR of 5 (*p* < 0.001). The 272 CFU represented 45 different bacterial species, with 38 species isolated in the control group and 21 in the intervention group. The most frequently isolated bacteria were *Cutibacterium acnes* (82/272, 30%), and *Staphylococcus epidermidis* (36/272, 13%). Of the 36 *S. epidermidis* isolates, 11 (31%) were drug-resistant, including three multidrug-resistant. One patient in the control group was infected by *Staphyloccocus aureus* and *Staphylococcus lugdunensis*, neither of which was isolated during the preparation of sterile goods. One patient in the intervention group developed an SSI caused by *C. acnes*, *Corynebacterium kroppenstedtii*, and *S. epidermidis*. *C. acnes* and *S. epidermidis* were isolated during the preparation.

**Conclusions:**

Minimising the number of personnel in the OR during preparation of sterile surgical goods is important to reduce the bacterial load.

**Trial registration:**

Prospectively 15 May 2022 at FoU Sweden (275659) and retrospectively 22 October 2022 at ClinicalTrials.Gov (NCT05597072).

## Introduction

Healthcare-associated infections are significant yet preventable patient safety concerns. Globally, approximately 43 million inpatients each year experience such infections, leading to increased morbidity and healthcare costs [1]. These infections represent a major challenge to patient safety, emphasising the need for effective infection control and prevention [2]. Surgical site infection (SSI) is the second most common type of healthcare-associated infection in both Europe and the USA [1]. Effective antimicrobial drugs are prerequisites for both preventive and curative measures, protecting patients and ensuring that complex surgical procedures can be provided at low risk [3]. In 2019, bacterial antimicrobial resistance was associated with 4.95 million deaths, and 1.27 million of these deaths were directly attributable to resistance [1]. SSIs contribute to prolonged hospital stays, which not only increase patient suffering but also result in higher healthcare costs due to greater occupancy of hospital beds and resources [4].

A widely used strategy for preventing SSIs is the implementation of specialised ventilation systems within the operating room (OR) [5]. These systems are designed to reduce the risk of airborne bacterial contamination by filtering the air, controlling its distribution, and diluting particles that could carry bacteria, thereby safeguarding sterile surgical goods and patients’ surgical wounds [6]. Additionally, ventilation systems should maintain positive air pressure inside the OR to prevent the influx of less clean air from outside the room [7].

Swedish guidelines recommend that sterile surgical goods should be prepared either within the OR before the patient enters, or in a preparation room directly adjacent to the OR [8]. The main source of bacterial air contamination is dispersion from the individuals present in the OR [9–11]. Previous studies have suggested that having more people present in the OR results in higher rates of bacterial air contamination [10, 12–14]. For example, one study found that 25–45% of the OR personnel dispersed methicillin-resistant *Staphylococcus epidermidis* into the air [10]. Given the growing concerns regarding antimicrobial resistance, it is crucial to optimise the conditions for patient safety [3]. We therefore aimed to investigate the effect of the number of personnel in the OR on bacterial air contamination during the preparation of sterile surgical goods. In addition, we performed species-level identification and antibiotic susceptibility testing of bacteria isolated within the OR and described the number of SSIs together with causative microorganisms.

## Methods

### Design and intervention

This randomised controlled trial investigated the preparation of sterile surgical goods for 70 open-heart surgeries. The inclusion criterion was a planned open-heart surgery for an adult, and the exclusion criterion was surgery on a patient with an existing infection. Cases were randomised into two groups: the intervention and the control, with the allocation sequence generated using sealed envelopes. The intervention group had the sterile surgical goods prepared by a maximum of two individuals within the OR, while the control group followed the standard procedure within the department. In the control group, the OR was prepared concurrently by various professionals, as routine. Specifically, while the OR nurses arranged the sterile surgical instruments, other professionals, including nurse anesthetists, perfusionists responsible for the heart-lung machine, and medical students, prepared their respective materials. This resulted in a mean of five individuals in the OR during preparation of the surgical goods in the control group (i.e., the number of individuals on each occasion was counted, summoned up to a total score and divided by the total number of occasions). Bacterial air contamination was passively collected on blood agar plates (blood agar medium 3.9% w/v [Colombia Blood Agar Base, Acumedia Neogen Corporation, Lansing, MI, USA] supplemented with 6% w/v defibrinated horse blood) and fastidious anaerobe agar (FAA) plates (LAB 90 FAA 4.6% w/v [LAB M Ltd., Lancashire, UK] supplemented with 5% w/v defibrinated horse blood). Blood agar plates were used to isolate aerobic bacteria, whereas FAA plates were used for the anaerobic bacteria.

The primary endpoint was bacterial contamination, measured as colony forming units (CFU). Other endpoints were descriptions of the different types of bacteria isolated in the OR, and SSI after two months.

### Settings and procedures

Data on bacterial air contamination within the OR were collected at the Department of Cardiothoracic and Vascular Surgery, Örebro University Hospital, Sweden, between May 2022 and March 2024. Data collection was either performed or supervised by the first author. The ORs were equipped with upward displacement ventilation, delivering air at a preset temperature of 19 °C and an inflow rate of approximately 700 L/sec. Prior to preparation, all flat surfaces in the OR were disinfected and the ventilation system was inspected. During both preparation and data collection, all personnel wore caps, face masks, and tightly woven reusable clothing. Additionally, the OR nurse preparing the sterile surgical goods wore a sterile, single-use surgical gown and double gloves. All wrapped sterile surgical goods were placed in the OR before data collection commenced. Three blood agar plates and three FAA plates were placed onto a separate sterile draped table for bacterial sampling. The tables holding the agar plates were consistently positioned at the center of the operating room (OR) on each occasion. The ORs maintained uniform interior configurations, with air inflow from the corners of the room and an outlet in the ceiling. Each OR had two entry points: one door opened to the corridor, whereas the other door, equipped with an airlock, led to an adjacent room with OR ventilation.

When the OR nurse entered the OR after surgical hand disinfection the data collection began by the assisting OR nurse opening the lids of the plates. The time and number of individuals present in the OR were recorded during data collection. The OR nurse and assisting nurse then continued to prepare the sterile surgical goods in a standardised manner. Throughout the data collection period, door openings and the number of individuals in the OR were continuously documented. Data collection ended when the preparation of sterile surgical goods was completed by closing the lids of the plates. Afterward, the plates were stored in a refrigerator until they were transported to the laboratory for analysis. All plates were analysed by laboratory personnel who were blinded to the allocation. Patient-reported symptoms of SSI were collected via telephone follow-up, using a simplified version of the ASEPSIS score [15].

### Microbiology

A total of 207 blood agar plates and 207 FAA plates were used to isolate bacterial air contamination. Each plate had a diameter of 9 cm (area: 63.6 cm^2^). The media for the plates were prepared by the Department of Laboratory Medicine at Örebro University Hospital, which is accredited by Swedac, the Swedish national accreditation body [16]. Quality control was performed on each batch of media before release. Upon arrival at the Department of Microbiology, the blood agar plates were incubated at 36 °C under aerobic conditions, while the FAA plates were incubated at 37 °C under anaerobic conditions (10% H_2_, 10% CO_2_, 80% N_2_). Bacterial growth was assessed by counting CFU per plate after 24 and 48 h of aerobic incubation, and after five days of anaerobic incubation. Bacterial isolates were identified using routine diagnostic methods, and species identification was performed using matrix-assisted laser desorption/ionization time-of-flight mass spectrometry (MALDI-TOF MS; Microflex and Biotyper 3.1, Bruker Daltonik, Bremen, Germany).

The disc diffusion test was used for antibiotic susceptibility testing of the staphylococci. The antibiotics tested were clindamycin (2 µg), erythromycin (15 µg), flucloxacillin (30 µg), fusidic acid (10 µg), gentamicin (10 µg), norfloxacin (10 µg), rifampicin (5 µg), and trimethoprim/sulfamethoxazole (25 µg). All antibiotic discs were obtained from Oxoid (Basingstoke, UK). A 0.5 McFarland bacterial suspension in 0.85% NaCl was applied to Mueller Hinton II agar plates (3.8% w/v, Oxoid) for testing. After 16–20 h of incubation at 35 °C, the zone diameters of inhibition were measured and interpreted according to the guidelines of the European Committee on Antimicrobial Susceptibility Testing [17]. Isolates resistant to at least three different antibiotic classes were classified as multidrug-resistant. The susceptibility of *Cutibacterium acnes* to benzylpenicillin, clindamycin, and vancomycin was assessed using a gradient test. Minimum inhibitory concentrations were determined using Etest (bioMérieux, Marcy l’Etoile, France) on FAA plates (LAB M) with a 0.5 McFarland bacterial suspension in NaCl and incubated at 36 °C in an anaerobic atmosphere for 24 h. For metronidazole susceptibility, a disc diffusion test was performed using discs from Oxoid.

### Statistical analysis

The primary aim was to determine whether the number of individuals present in the OR during the preparation of sterile surgical goods influenced the air quality. A formal power calculation was not conducted because of the lack of data on the expected contamination in this study design. However, after making comparison with an earlier study using a similar set-up, 70 occasions were considered sufficient for the present analysis [18]. Statistical analysis was performed using version 29 of SPSS (SPSS Statistics, IBM, Armonk, NY, USA). Bacterial counts and other non-normally distributed variables were analysed using the Mann-Whitney *U* test, continuous data were analysed using Student’s *t*-test, and categorical variables were analysed using either the chi-square test or Fisher’s exact test, depending on the data. Descriptive statistics included mean, median, number, percentage, confidence interval, interquartile range, and standard deviation. A two-tailed *p*-value of < 0.05 was considered statistically significant.

## Results

**Fig. 1:**
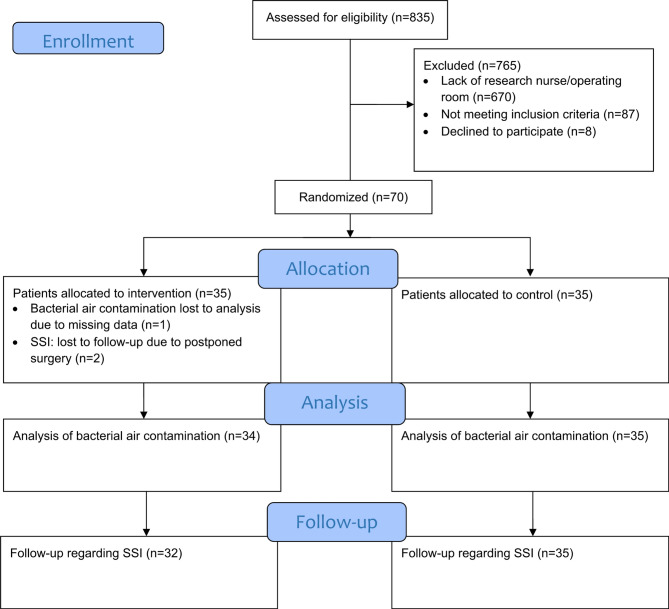
Flowchart of the inclusion process regarding bacterial air contamination and surgical site infections (SSIs)

Of the 70 surgeries included in the study, three in the intervention group did not proceed as originally planned. One patient’s data was excluded because of the use of sterile surgical goods in an unplanned emergency procedure during the night, leading to that the plates for data collection were compromised. In the other two cases, the surgeries were rescheduled to later dates, but the plates from the preparation with the bacterial air contamination data were still collected (Fig. 1). This led to the missing of one occasion of bacterial contamination analysis and three follow-up occasions of SSI.

All surgeries included in the study were open-heart surgeries, with the most common being coronary artery bypass grafting (*n* = 31) followed by aortic valve replacement (*n* = 11). Bacterial growth was assessed using 207 blood agar plates and 207 FAA plates. The mean time required to prepare the sterile surgical goods for open-heart surgery was 32 min (SD: 9.3). Characteristics of the patients, surgeries, and OR are detailed in Table 1.

**Table 1:** Characteristics of patients, open-heart surgeries, and operating room during Preparation of sterile surgical goods

Characteristics	Control *n* = 35 33 men, 2 women	Intervention *n* = 34^a^ 29 men, 5 women	*p*-value
**Patients**			
Age years, mean (SD)	68.1 (8.26)	68.2 (10.6)	0.950
BMI, mean (SD)	26.7 (3.9)	29 (3.77)	0.015
Eczema, *n* (%)	4 (11.4)	2 (5,7)	0.672
Diabetes, *n* (%)	11 (31.4)	9 (25.7)	0.795
**Data collection**			
Preparation time min, mean (SD)	31.5 (5.1)	33.2 (4.8)	0.158
Individuals during preparation, mean (SD)	4.62 (1.1)	2.02 (0.17)	0.001
Door openings, corridor, median (IQR)	0 (0)	0 (0)	0.066
Door openings, air lock, median (IQR)	5.5 (4)	0 (1)	0.001
**Surgery**			
CABG, *n* (%)	17 (48.5)	14 (41)	
AVR, *n* (%)	6 (17.1)	5 (14.7)	
Ascending aortic aneurysm repair, *n* (%)	6 (17.1)	5 (14.7)	
MI, *n* (%)	3 (8.6)	3 (8.9)	
CABG and AVR, *n* (%)	1 (2.9)	5 (14.7)	
CABG and MI, *n* (%)	1 (2.9)	1 (3)	
AVR and MI, *n* (%)	0	1 (3)	
Other, *n* (%)	1 (2.9)	0	

^a^ One missing patient in this group. AVR = aortic valve replacement, BMI = body mass index, CABG = coronary artery bypass graft, IQR = interquartile range, MI = mitral valve insufficiency, SD = standard deviation. Continuous variables were analysed using Student’s *t*-test. Non-normal distributed data used Mann Witney U test and Fisher’s exact test A two-tailed *p*-value < 0.05 was considered statistically significant

**Fig. 2:**
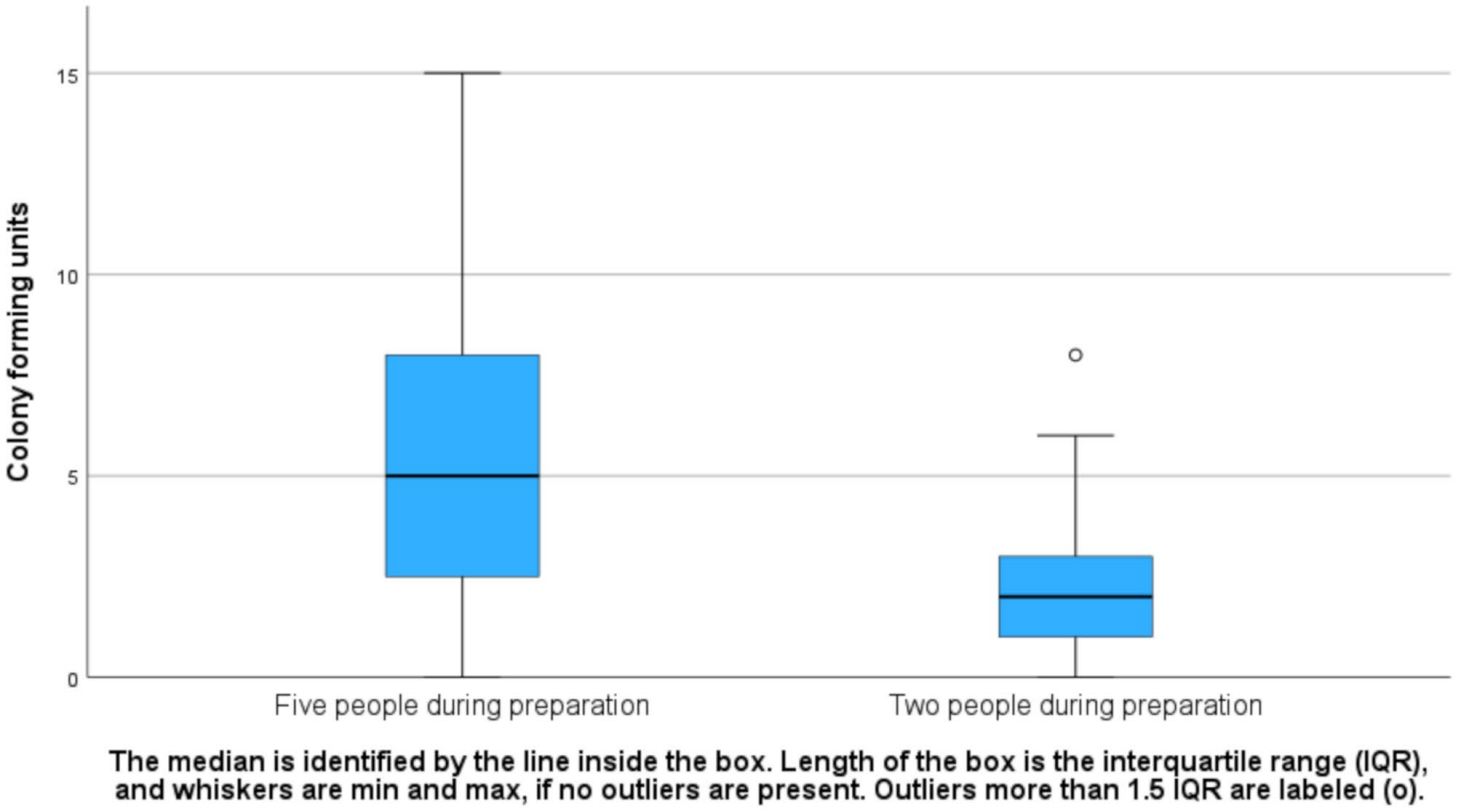
Bacterial air contamination during preparation of sterile surgical goods within an operating room, divided by the number of personnel present (two vs. five)

The total bacterial air contamination in the OR, as isolated on both blood agar and FAA plates, amounted to 272 CFU in both groups, comprising 199 (73%) from the control group and 73 (27%) from the intervention group. When sterile surgical goods were prepared with two personnel, the median CFU in the intervention group was 2 with an IQR of 2, and in the control group with five personnel, the median CFU was 5 with an IQR of 5. Statistical analysis revealed a significant difference, with less bacterial air contamination observed when two individuals were present during preparation of sterile surgical goods (intervention group) compared to five individuals (control group) (*p* < 0.001; Fig. 2). The proportion of contaminated versus non-contaminated plates was 73/204 (36%) in the intervention group and 101/210 (48%) in the control group (*p* = 0.45).

The 272 CFU found during the preparation comprised 45 different bacterial species, with 38 species isolated in the control group and 21 species in the intervention group (Table 2). In descending order, the most frequently isolated bacteria were *Cutibacterium acnes* (82/272, 30%), *S. epidermidis* (36/272, 13%), *Micrococcus luteus* (35/272, 13%), *Staphylococcus hominis* (20/272, 7%), and *Staphylococcus capitis* (18/272, 7%), accounting for 70% of the total bacterial isolates. Other bacterial species were also found in smaller quantities. *S. aureus* was isolated twice in the control group, with a total of 2 CFU (Table 2).

**Table 2:** Microorganisms isolated in the operating room (OR) during Preparation of sterile goods for 69 open-heart surgeries

	Number of colony forming units		
	*Control*	*Intervention*	Total
	Five individuals in the OR	Two individuals in the OR	
*Bacillus cereus*		1	1
*Brachybacterium muris*	4		4
*Corynebacterium pseudodiphtheriticum*	1		1
*Corynebacterium sanguinis*	1		1
*Corynebacterium sp.*	3		3
*Corynebacterium xerosis*	1		1
*Cutibacterium acnes*	47	35	82
*Dermabacter sp.*		1	1
*Dermacoccus sp.*	1		1
*Dermacoccus nishinomiyaensis*	1		1
*Dietzia sp.*	1		1
*Enterococcus casseliflavus*	1		1
*Gemella haemolysans*	1		1
Gram-negative rods	1		1
Gram-positive cocci	3		3
Gram-positive rods	3	2	5
*Kocuria sp.*	1		1
*Kytococcus sp.*		1	1
*Microbacterium sp.*	1		1
*Micrococcus flavus*	1	1	2
*Micrococcus luteus*	32	3	35
*Micrococcus lylae*	1		1
*Micrococcus sp.*	8	2	10
*Micrococcus terreus*	1		1
*Moraxella osloensis*	2	2	4
*Paenibacillus sp.*	1		1
*Peptoniphilus sp.*	1		1
*Pseudoclavibacter alba*		1	1
*Rothia dentocariosa*		1	1
*Rothia koreensis*	1		1
*Sphingobacterium multivorum*	1		1
*Staphylococcus aureus*	2		2
*Staphylococcus capitis*	16	2	18
*Staphylococcus caprae*	1	1	2
*Staphylococcus cohnii*	2	1	3
*Staphylococcus epidermidis*	26	10	36
*Staphylococcus haemolyticus*	2		2
*Staphylococcus hominis*	17	3	20
*Staphylococcus lugdunensis*	2	2	4
*Staphylococcus petrasii*	2	1	3
*Staphylococcus saprophyticus*	2		2
*Staphylococcus warneri*	6	1	7
*Streptococcus mitis*		1	1
*Streptococcus urinalis*		1	1
*Streptomyces sp.*	1		1
Total	199	73	272

Total number of microorganisms isolated on aerobe and anaerobe blood agar plates (*n* = 414) during 69 surgeries. Each plate was 9 cm in diameter (area: 63.6 cm^2^).

Both *S. aureus* isolates were susceptible to all the eight antibiotics tested. Of the 36 *S. epidermidis* isolates, 31% (11/36 CFU) were drug-resistant, and three of these were multidrug-resistant. Seven of the drug-resistant *S. epidermidis* isolates came from the control group, and four from the intervention group (Table 3). The *C. acnes* isolates were susceptible to all antibiotics tested, except for one isolate that demonstrated resistance to vancomycin.

**Table 3:** Antibiotic susceptibility of 36 drug-resistant *Staphylococcus epidermidis* isolates collected during Preparation of sterile surgical goods

Antibiotic susceptibility of S. epidermidis on every occasion	Antibiotics tested							
	CN10	DA	E	FD	FOX	NOR	RD	SXT
1 CFU occasion 2, C				R				R
1 CFU occasion 17, C				R				
1 CFU occasion 29, C*		R	R		R			
1 CFU occasion 34, C			R					
1 CFU occasion 45, C			R					
1 CFU occasion 47, C*		R	R	R				R
1 CFU occasion 70, C				R				
1 CFU occasion 16, I			R	R				
1 CFU occasion 27, I						R		
1 CFU occasion 39, I			R					
1 CFU occasion 58, I*		R			R			R

CFU = colony forming units. C = control, I = intervention, R = drug resistance to that specific antibiotic. *Multidrug resistant (resistant to ≥ 3 antibiotics). CN10 = gentamicin, DA = clindamycin, E = erythromycin,

FD = fusidic acid, FOX = flucloxacillin, NOR = norfloxacin, RD = rifampicin, SXT = trimethoprim and sulfamethoxazole

Two patients developed SSIs within two months, one in each group. The patient in the control group was infected with *S. aureus* and *Staphylococcus lugdunensis*, neither of which was isolated during the preparation of that patients’ sterile surgical goods. The bacteria isolated during the preparation were *C. acnes* (*n* = 4 CFU), *M. luteus* (*n* = 2 CFU), and *Staphylococcus warneri* (*n* = 1 CFU). The patient in the intervention group developed an SSI caused by *C. acnes*, *Corynebacterium kroppenstedtii*, and *S. epidermidis. C. acnes* (*n* = 1 CFU) and *S. epidermidis* (*n* = 2 CFU) were isolated during the preparation of the sterile surgical goods.

## Discussion

Swedish national guidelines recommend that sterile surgical goods should be prepared before the patient enters the OR, or in a preparation room adjacent to the OR [8]. Nevertheless, there has been a long-standing debate regarding whether the goods can be prepared in the OR while the patient is already present and being readied for anaesthesia. This debate likely arises from a desire for time efficiency and a lack of conclusive evidence contraindicating the practice. Our results indicate that an increased number of personnel in the OR during the preparation of sterile surgical goods caused a higher level of bacterial air contamination. To minimise bacterial air contamination during the preparation phase, the number of personnel in the OR should be as low as possible, as all individuals disperse bacteria into the air. This finding is consistent with previous studies showing an association between the number of personnel in the OR and an increase in CFU in the air during orthopaedic surgery [12, 19]. The results of our study indicated that bacterial spread within the OR increased with the number of individuals present. Consequently, the distribution of drug-resistant bacteria also increased. These findings underscore the importance of minimizing the number of individuals in the OR to reduce bacterial air contamination.

The total bacterial air contamination found in our study was 272 CFU across all 69 sessions of preparation for open-heart surgeries. This suggests that the impact of bacterial air contamination may be relatively small compared to the contribution from other possible sources of bacterial contamination. The low number of CFU found dispersed from the personnel during preparation may be considered low compared to the patient’s residual skin flora following surgical skin disinfection. Surgical wounds can be contaminated by the patient’s skin flora, and so skin disinfection prior to surgery is intended to sterilise the skin. Although the skin most likely does not become or remain entirely sterile [20–23], disinfection substantially reduces the number of bacteria present [21]. Nevertheless, almost 30–50% [22, 24] of patients have some number of bacteria remaining on the skin, with a wide range from a single isolated CFU up to 5,000 CFU or more [20, 24].

The Swedish ISO standard recommends both active and passive assessment of air quality in the OR; however, this standard was not designed for various research methodologies [25]. Specific research designs often lack standardization in interventions and sampling methods. The design for the intervention group was to optimize conditions and involved only two individuals, reflecting a possible real-life working scenario. This optimal conditions with only two individuals would be compared with our present situation with almost always more individuals within the OR. Active data collection required a third person to operate the air sampling machine, making it less suitable than passive agar plates. The decision to use passive data collection was based on the study design and the properties of the two available agar plates. The target bacteria were not environmental but common bacteria dispersed by humans within the OR. Blood agar plates and FAA plates were chosen because they demonstrate the growth of both aerobic and anaerobic bacteria, making them an optimal set-up.

One limitation was the relatively short data collection period of approximately 30 min, whereas the SIS recommends a duration of 60 min [25]. However, the preparation of sterile surgical goods rarely exceeds 60 min, but should be considered when interpreting the results. The positioning of the table with agar plates may have influenced the contamination rate. To control this potential effect, the tables with the agar plates were consistently placed in the center of the OR on each occasion, away from the doors and air inflow from the corners. If the table had been positioned in the corners near the air inflow, the agar plates might have been less contaminated, resulting in less reliable data. Conversely, if the plates had been placed near the doors, they might have been more contaminated because of the turbulent air caused by door movements. The origin of the bacteria causing SSI and those isolated during preparation were not analyzed by genome sequencing. Consequently, the specific bacteria responsible for the patients’ SSI remain unidentified. We deemed it reasonable to include SSI descriptively in this study, acknowledging that the study was not designed to detect differences in SSI.

The likelihood of developing an SSI is influenced by both the bacterial load and the virulence of the contaminating bacterium. Our study identified a range of different bacteria with varying virulence; from the slow-growing *C. acne* and *S. epidermidis*, both potential causes of SSI, to *S. aureus*, one of the most virulent bacteria [26]. In addition to bacterial factors, the patient’s immune system also plays a critical role in determining the outcome. Immunocompromised patients have a weakened resistance to bacteria, making them more susceptible to SSIs even when exposed to low-virulence bacteria such as *S. epidermidis* [27, 28]. *S. epidermidis* was the second most common bacterium isolated in the present study and is a well-known component of the human skin flora [29]. We found that 31% of the *S*. *epidermidis* isolates were resistant. Furthermore, we identified only two *S. aureus* isolates, which could be considered a low number. An increased number of individuals is likely to increase the bacterial count, suggesting a higher probability of encountering drug-resistant bacteria. Evidence suggests that air quality depends not only on the ventilation system and clothes worn, but also on OR size [30, 31] in relation to the number of personnel present [13, 32–34].

Another limitation was the lack of control over door openings between the groups. The control group experienced significantly more door openings with airlocks compared to the intervention group. Theoretically, the use of doors with airlocks was believed to mitigate the impact of a positive air pressure. Furthermore, limitation was that we did not analyse how movement by personnel affected air quality, and we did not investigate the correlation between increased bacterial air contamination and the development of SSIs. Therefore, future research is needed to explore the relationship between bacterial air contamination and SSIs to better understand how airborne bacteria contribute to SSIs.

## Conclusion

The presence of five individuals involved in the preparation of sterile surgical goods, as opposed to two, resulted in a significantly higher level of bacterial air contamination in the OR. These findings underscore the importance of limiting the number of personnel in the OR to minimise general bacterial contamination.

## Data Availability

The datasets used and analysed in this study are available from the corresponding author on reasonable request.
